# Antibiotic susceptibility testing of *Mycoplasma hyopneumoniae* field isolates from Central Europe for fifteen antibiotics by microbroth dilution method

**DOI:** 10.1371/journal.pone.0209030

**Published:** 2018-12-11

**Authors:** Orsolya Felde, Zsuzsa Kreizinger, Kinga Mária Sulyok, Veronika Hrivnák, Krisztián Kiss, Ákos Jerzsele, Imre Biksi, Miklós Gyuranecz

**Affiliations:** 1 Institute for Veterinary Medical Research, Centre for Agricultural Research, Hungarian Academy of Sciences, Budapest, Hungary; 2 SCG Diagnosztika Kft., Délegyháza, Hungary; 3 Department of Pharmacology and Toxicology, University of Veterinary Medicine, Budapest, Hungary; 4 Department and Clinic of Production Animals, University of Veterinary Medicine, Üllő, Hungary; 5 Department of Microbiology and Infectious Diseases, University of Veterinary Medicine, Budapest, Hungary; Ross University School of Veterinary Medicine, SAINT KITTS AND NEVIS

## Abstract

*Mycoplasma hyopneumoniae* infections are responsible for significant economic losses in the swine industry. Commercially available vaccines are not able to inhibit the colonisation of the respiratory tract by *M*. *hyopneumoniae* absolutely, therefore vaccination can be completed with antibiotic treatment to moderate clinical signs and improve performances of the animals. Antibiotic susceptibility testing of *M*. *hyopneumoniae* is time-consuming and complicated; therefore, it is not accomplished routinely. The aim of this study was to determine the *in vitro* susceptibility to 15 different antibiotics of *M*. *hyopneumoniae* isolates originating from Hungarian slaughterhouses and to examine single-nucleotide polymorphisms (SNPs) in genes affecting susceptibility to antimicrobials. Minimum inhibitory concentration (MIC) values of the examined antibiotics against 44 *M*. *hyopneumoniae* strains were determined by microbroth dilution method. While all of the tested antibiotics were effective against the majority of the studied strains, high MIC values of fluoroquinolones (enrofloxacin 2.5 μg/ml; marbofloxacin 5 μg/ml) were observed against one strain (MycSu17) and extremely high MIC values of macrolides and lincomycin (tilmicosin, tulathromycin and lincomycin >64 μg/ml; gamithromycin 64 μg/ml; tylosin 32 μg/ml and tylvalosin 2 μg/ml) were determined against another, outlier strain (MycSu18). Amino acid changes in the genes *gyrA* (Gly81Ala; Ala83Val; Glu87Gly, according to *Escherichia coli* numbering) and *parC* (Ser80Phe/Tyr; Asp84Asn) correlated with decreased antibiotic susceptibility to fluoroquinolones and a SNP in the nucleotide sequence of the 23S rRNA (A2059G) was found to be associated with increased MIC values of macrolides. The correlation was more remarkable when final MIC values were evaluated. This study presented the antibiotic susceptibility profiles of *M*. *hyopneumoniae* strains circulating in the Central European region, demonstrating the high *in vitro* efficacy of the tested agents. The observed high MIC values correlated with the SNPs in the examined regions and support the relevance of susceptibility testing and directed antibiotic therapy.

## Introduction

*Mycoplasma hyopneumoniae* is a member of the class *Mollicutes* [1] and the causative agent of porcine enzootic pneumonia [2]. *M*. *hyopneumoniae* infection affects especially growing pigs, causing significant economic losses in the swine industry worldwide, by chronic cough, growth retardation and predisposing animals to secondary infections [3–6]. Improvement of the management system and the environmental conditions of pig farms are essential parts of the control strategies just like vaccination and antibiotic treatment [4,7,8]. As vaccination alone is not always effective enough to prevent colonisation of the respiratory tract [9], antibiotic treatment might be necessary [4].

*Mycoplasmas* are resistant to antimicrobials that interfere with folic acid metabolism and cell wall synthesis, like sulphonamides, trimetoprim and the β-lactam class of antibiotics [5,10]. Macrolides, tetracyclines, fluoroquinolones, some aminoglycosides and aminocyclitols, lincosamides and pleuromutilins are active antimicrobial agents against *M*. *hyopneumoniae* [4,11]. However, studies have already drawn attention on the emergence of antibiotic resistance in *M*. *hyopneumoniae* to fluoroquinolones, macrolides, lincosamides and tetracyclines [12–14]. The decreased susceptibility may appear as a consequence of excessive medication [13,15,16]. The basics of *in vitro* susceptibility testing with microbroth dilution method were laid down almost 20 years ago [17], however, important points of standardisation are still absent.

Genomic changes (e.g. single-nucleotide polymorphism (SNP)) related to decreased effectiveness of certain antibiotics have been identified in previous publications [14,15,18,19]. Fluoroquinolones and macrolides are among the most frequently utilised antibiotic agents to control *M*. *hyopneumoniae* infection in Hungary [20,21]. The targets of the fluoroquinolone type antibiotics enrofloxacin and marbofloxacin, are topoisomerase enzymes (DNA gyrase and topoisomerase IV), which have essential role in bacterial DNA replication [15,18]. Emerging resistance to fluoroquinolones in mycoplasmas is usually due to transitions in the quinolone resistance-determining regions (QRDR) in genes encoding subunits of the topoisomerase enzymes (*gyrA*, *gyrB*, *parC*, *parE*) [19,22]. The majority of the substitutions, causing amino acid change and therefore increased MIC values, are observed in the *parC* gene (e.g. Ser80Phe, Ser80Tyr, Asp84Asn and Ala116Glu, according to *Escherichia coli* numbering) [15,18]. The amino acid change Ala83Val in *gyrA* gene was also described to be related to decreased susceptibility to enrofloxacin in *M*. *hyopneumoniae* [18]. Further amino acid substitutions in the *gyrA* gene observed in other *Mycoplasma* species were for example Gly81Ala or Glu87Gly [23,24]. According to the literature, 14-membered macrolides show low MIC against *M*. *hyopneumoniae* due to a G2057A transition in the 23S rRNA sequence [14]. In addition, adenosine→guanosine transition at nucleotide 2058 in the same region is frequently observed in association with increased resistance to 15- and 16-membered macrolides and lincosamides [14,25].

The aim of this study was to describe the antibiotic susceptibility profile of 44 *M*. *hyopneumoniae* strains isolated from Hungarian slaughterhouses in years 2015 and 2016, against 15 antimicrobial agents and to examine the genetic background of increased MIC values.

## Materials and methods

### Sample collection

Forty-four *M*. *hyopneumoniae* strains originating mainly from Hungary (n = 40), but also from Slovakia (n = 3) and the Czech Republic (n = 1) were tested in this study (S1 and S2 Tables). Hungarian slaughterhouses were visited between 2015 and 2016 for sampling. Ethical approval and specific permission were not required for the study as all affected porcine lung samples, used for the isolation, were collected by the authors with the consent of the owners during routine diagnostic examinations of the carcasses in slaughterhouses. The affected lung samples were washed into Friis broth [26], and filtered through a 0.45 μm filter. The broth was diluted 30-fold and incubated for 4 weeks or until colour change at 37 °C. After the incubation period a 10-fold serial dilution was prepared, and incubated until colour change [27]. When colour change of the broth media occurred cultures were inoculated onto solid media and incubated at 37 °C and 5% CO_2_ for 4–10 days, until visible colonies appeared. *Mycoplasma* strains were once filter-cloned, and DNA extraction was performed from the pure cultures using QIAamp DNA mini kit (Qiagen Inc., Hilden, Germany) according to the manufacturer’s instructions. Species-specific PCR test was accomplished to confirm the presence of *M*. *hyopneumoniae* [28]. To exclude the presence of other *Mycoplasma* species sequence analyses and BLAST search were carried out using the amplicons of a universal *Mycoplasmatales* PCR system targeting the 16S/23S rRNA intergenic spacer region [29]. PCR products were sequenced on ABI 3130XL genetic analyser (Applied Biosystems, Foster City, CA). Aliquots of purified cultures were stored frozen at -70 °C until usage.

### Antibiotic susceptibility testing

The number of colour changing units (CCU) was determined by microbroth dilution method after four weeks of incubation [17]. Antimicrobial agents frequently used in Hungary [21] were selected for susceptibility tests: fluoroquinolones (enrofloxacin, marbofloxacin), aminoglycosides (gentamicin), aminocyclitols (spectinomycin), tetracyclines (oxytetracycline, doxycycline), macrolides (tylosin, tilmicosin, tylvalosin, tulathromycin, gamithromycin), pleuromutilins (tiamulin, valnemulin), phenicols (florfenicol) and lincosamides (lincomycin). Tylvalosin originated from ECO Animal Health Ltd., UK (Aivlosin), tulathromycin originated from Pfizer Inc., USA, and the rest of the products originated from VETRANAL, Sigma-Aldrich, Germany. The antibiotics were diluted and stored according to the recommendation of Hannan [17]. Stock solutions of 1 mg/ml were prepared in sterile distilled water, except the fluoroquinolones, tulathromycin, gamithromycin and florfenicol. Stock solutions of 1 mg/ml enrofloxacin and marbofloxacin were prepared in 0.1 M NaOH and stock solutions of 1 mg/ml tulathromycin, gamithromycin and florfenicol were prepared in 96% ethanol and sterile distilled water. Aliquots were stored at -70 °C until required, precipitation on thawing was checked before usage and dilutions for each test were freshly prepared. Twofold dilutions were made in the range 0.039–10 μg/ml for fluoroquinolones, pleuromutilins and doxycycline; 0.25–64 μg/ml for macrolides, gentamicin, spectinomycin, lincomycin and oxytetracycline; 0.125–32 μg/ml for florfenicol. Microbroth dilution test was accomplished using a 96-wells microtiter plate, containing growth control (bacterium culture in broth media), sterility control (broth media without bacterium culture) and end point control (sterile broth media adjusted to pH 6.8). By reason of the more pronounced colour change of the media, Mycoplasma Experience broth medium (Mycoplasma Experience Ltd., Bletchingley, United Kingdom) was applied for determining the number of CCU of strains and the susceptibility tests. The antibiotic susceptibility test was accomplished on 10^4^−10^5^ CCU/ml of the strains as recommended by Hannan [17]. All strains were tested in duplicates and all plates contained a duplicate of the type strain (NCTC 10110) as a quality control. MIC was established as the lowest antibiotic concentration where no colour change of the broth was observed as a consequence of the absence of bacterial metabolism. Initial MIC values were recorded when colour change of the broth media of the growth control was visible (4–14 days after inoculation) (S1 Table), and final MIC values were registered when no further colour change was observed (S2 Table). MIC_50_ and MIC_90_ values were determined as the lowest concentrations that inhibited the growth of 50% or 90% of the strains [17].

### Sequence analysis

Genetic markers correlating with antibiotic susceptibility in *M*. *hyopneumoniae* were examined in genes *gyrA*, *gyrB*, *parC*, *parE* and 23S rRNA [14,15,18,19]. While for the amplification of the genes *gyrA* and *gyrB* primers and heat profile were used according to Vicca *et al*. [18], for the amplification of genes *parC* and *parE*, primers and heat profile were used according to Le Carrou *et al*. [15], with modification of the annealing temperature to 56 °C. For the analysis of the 23S rRNA sequence the PCR conditions of Stakenborg *et al*. [14] were used with some modification of the annealing temperature to 56 °C and the following forward (5’ GAT GAG TAT TCT AAG GTG AGC GAG 3’) and reverse (5’ CAG TCA AAC TAC CCA CCA CG 3’) primers. PCR products were sequenced on ABI 3130XL genetic analyser (Applied Biosystems, Foster City, CA) and sequence analysis was performed by using Geneious software 10.2.3 (Biomatters Ltd.) [30]. The validity of SNPs was confirmed by manual examination of the assembled sequences. Numbering of nucleotide and amino acid positions is based on genes and proteins of *Escherichia coli* strain K-12 substrain MG1655 (GenBank accession number CP014225). Susceptibility profiles and correlating genetic markers were evaluated in relation with previously determined genotypes of the examined strains also [31].

## Results

### Antibiotic susceptibility profiles

The initial MIC values are evaluated and discussed throughout the study [17], however, differences were registered between initial and final MIC values in certain cases (S1 and S2 Tables). MIC values of the studied antimicrobial agents against the type strain (NCTC 10110) were consistent throughout the study (Table 1), and these results were mostly in accordance with previously defined values gained by microbroth dilution method (enrofloxacin 0.015–0.2 μg/ml, marbofloxacin 0.031 μg/ml, oxytetracycline 0.12–1 μg/ml, gentamicin 0.25–5 μg/ml, tylosin ≤0.015–0.06 μg/ml, tylvalosin 0.06 μg/ml, lincomycin 0.05–0.125 μg/ml, tiamulin 0.008–0.125 μg/ml, valnemulin ≤0.001–0.008 μg/ml) [11–13,32–34]. However, minor differences (two-fold increase or decrease) were observed in the MIC values against the type strain compared to earlier data in case of doxycycline (0.06–0.5 μg/ml), spectinomycin (0.5 μg/ml), tilmicosin (0.25–1 μg/ml) and florfenicol (0.25–0.5 μg/ml) [13,32–34]. Moreover, the MIC value of tulathromycin was noticeably higher (10^3^ difference between MIC values) than that reported in the literature (≤0.001–0.002 μg/ml) [34]. Previously published MIC values for gamithromycin were not available at the time of the present study. The MIC ranges, the MIC_50_ and MIC_90_ values of each antibiotic against the examined strains, are recorded in Table 1.

**Table 1:** MIC values against the type strain and summary of MIC ranges, MIC_50_ and MIC_90_ values (μg/ml) against the *M*. *hyopneumoniae* strains involved in this study.

	NCTC 10110 initial	NCTC 10110 final	Range initial	Range final	MIC_50_ initial	MIC_50_ final	MIC_90_ initial	MIC_90_ final
**Fluoroquinolones**								
Enrofloxacin	≤0.039	0.078	≤0.039–2.5	≤0.039–5	≤0.039	0.312	1.25	2.5
Marbofloxacin	≤0.039	0.156	≤0.039–5	≤0.039–10	≤0.039	1.25	2.5	5
**Tetracyclines**								
Oxytetracycline	≤0.25	4	≤0.25–4	0.5–32	≤0.25	4	2	16
Doxycycline	≤0.039	0.625	≤0.039–0.625	0.078–2.5	0.078	0.625	0.312	2.5
**Aminoglycoside**								
Gentamicin	≤0.25	1	≤0.25–0.5	0.5–2	≤0.25	1	0.5	2
**Aminocyclitol**								
Spectinomycin	1	4	≤0.25–4	1–8	2	4	2	4
**Macrolides**								
Tylosin	≤0.25	0.5	≤0.25–32	≤0.25–64	0.25	0.5	≤0.25	0.5
Tilmicosin	2	8	≤0.25-≥64	2->64	2	8	4	16
Tylvalosin	≤0.25	≤0.25	≤0.25–2	≤0.25–8	≤0.25	≤0.25	≤0.25	≤0.25
Gamithromycin	1	4	≤0.25–64	1->64	0.5	4	2	8
Tulathromycin	1	4	≤0.25-≥64	0.5->64	0.5	2	1	4
**Lincosamide**								
Lincomycin	≤0.25	1	≤0.25-≥64	≤0.25->64	≤0.25	0.5	≤0.25	1
**Pleuromutilins**								
Tiamulin	≤0.039	0.156	≤0.039–0.156	0.078–0.312	≤0.039	0.156	0.078	0.156
Valnemulin	≤0.039	≤0.039	≤0.039	≤0.039	≤0.039	≤0.039	≤0.039	≤0.039
**Phenicol**								
Florfenicol	1	2	≤0.125–2	1–4	1	2	2	4

As official breakpoints of antibiotics against *M*. *hyopneumoniae* are not standardized, MIC values were compared to previously published, unofficial breakpoints [11] in the present study. No correlation was found between antibiotic susceptibility profiles and earlier assigned genotypes of the examined strains [31].

**Fig 1:**
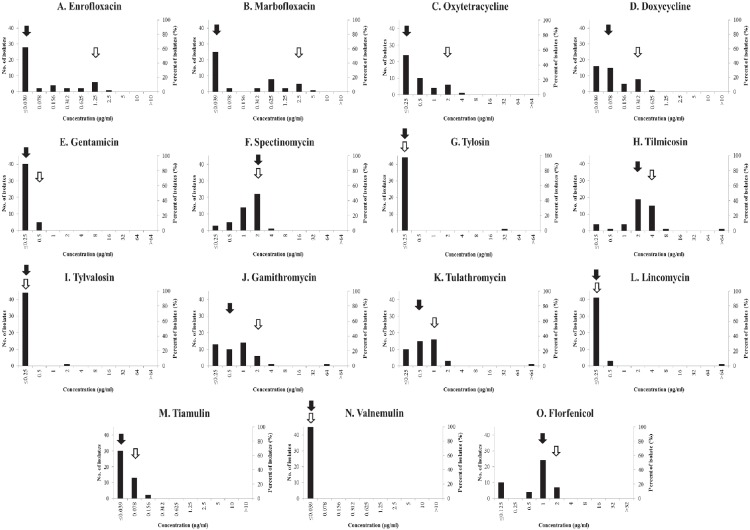
Distribution of the minimal inhibitory concentrations of each tested antibiotic against the studied *M*. *hyopneumoniae* isolates. MIC_50_ and MIC_90_ values are marked with black and white arrows, respectively.

The distribution of the MIC values of fluoroquinolones (enrofloxacin and marbofloxacin) showed one main peak coinciding with MIC_50_ value at the lowest antibiotic concentration (≤0.039 μg/ml), while the other values represented equipartition with the highest MIC values (2.5 μg/ml and 5 μg/ml, respectively) (Fig 1A and 1B). One strain (MycSu17) exceeded the unofficial breakpoint [11], with the MIC value of 2.5 μg/ml of enrofloxacin. All of the examined tetracyclines had low MIC values with MIC_50_ and MIC_90_ values of ≤0.25 μg/ml and 2 μg/ml of oxytetracycline; and 0.078 μg/ml and 0.312 μg/ml of doxycycline (Fig 1C and 1D). The lowest examined concentration of gentamicin (≤0.25 μg/ml) was effective against most of the studied strains (Fig 1E). MIC_50_ and MIC_90_ values of spectinomycin were 2 μg/ml, with MIC 4 μg/ml being the highest detected value (Fig 1F). Five macrolides were tested (Fig 1G–1K), out of which tilmicosin showed a Gaussian distribution with 2 μg/ml and 4 μg/ml MIC_50_ and MIC_90_ values, respectively. One main peak at the lowest antibiotic concentration (≤0.25 μg/ml) was observed in the MIC values of tylosin and tylvalosin against the examined strains. MIC_50_ values of gamithromycin and tulathromycin were 0.5 μg/ml, while MIC_90_ values were 2 μg/ml and 1 μg/ml, respectively. Both MIC_50_ and MIC_90_ values of lincomycin coincided with the lowest examined antibiotic concentration (≤0.25 μg/ml) (Fig 1L). For all macrolides and for lincomycin high MIC values (>64 μg/ml of tilmicosin and tulathromycin; 64 μg/ml of gamithromycin; 32 μg/ml of tylosin; 2 μg/ml of tylvalosin; and >64 μg/ml of lincomycin) were detected against an outlier strain (MycSu18). Both studied pleuromutilins had low MIC values (Fig 1M and 1N). The MIC_50_ and MIC_90_ values of valnemulin were ≤0.039 μg/ml, while that of tiamulin ≤0.039 μg/ml and 0.078 μg/ml. MIC_50_ and MIC_90_ values of florfenicol were 1 μg/ml and 2 μg/ml (Fig 1O).

### Single-nucleotide polymorphisms correlating with decreased antibiotic susceptibility

High MIC values of fluoroquinolones, macrolides and lincosamides, exceeding the unofficial breakpoints [11] were found in some cases (e.g. MycSu17-18). Both synonymous and non-synonymous substitutions were observed in genes associated with susceptibility to fluoroquinolones (*gyrA*, *gyrB*, *parC* and *parE*); however, only SNPs resulting in amino acid alterations were further examined in the present study. None of the amino acid changes in the genes *gyrB* and *parE* showed correlation with the defined MIC values. On the other hand, amino acid changes in the *gyrA* gene (Gly81Ala, Ala83Val and Glu87Gly) and in the *parC* gene (Ser80Phe, Ser80Tyr or Asp84Asn) correlated with decreased susceptibility of fluoroquinolones (S3 Table). Single alterations in the *parC* gene seem to have no crucial effect on fluoroquinolone susceptibility when initial MIC values are examined. On the other hand, at least 12-fold concentration difference is observed in the final MIC values against strains, which contain a single alteration in the *parC* gene. As opposed to the observed slight increase of MIC values of fluoroquinolones in association with the single substitution event in gene *parC*, double substitutions in genes *parC* and *gyrA* correlated with final MIC values higher than 2 μg/ml in all cases, with one exception (MycSu44). It is noteworthy, that the double substitutions in strain MycSu44 consisted of Ala83Val in gene *gyrA* and Asp84Asn in *parC*, while the rest of the strains showed various amino acid substitution types in gene *gyrA* but only the change of serine at amino acid position 80 in gene *parC*. The one outlier strain (MycSu17) against which 2.5–5 μg/ml initial MIC values of fluoroquinolones were detected contained the double substitution combination Ser80Phe (in *parC* gene) with Ala83Val (in *gyrA* gene). Correlation was described between increased MIC values of macrolides and lincosamides against Mycoplasma species/ *M*. *hyopneumoniae* and SNPs in the 23S rRNA sequence [14,19]. A nucleotide substitution at the position A2059G was found in the outlier strain (MycSu18) showing extremely decreased susceptibility to macrolides and lincosamides (S3 Table). The observed SNPs in the strains originating from the same herds were consistent with one exception: the strains originating from Mezőtúr (MycSu7; 8 and 41), which also clustered into completely different sequence types according to earlier genotyping analysis [31] showed distinct susceptibility profiles and genetic alterations correlating with antibiotic susceptibility.

## Discussion

Antibiotic susceptibility testing of porcine mycoplasmas is not performed routinely, because it is fastidious, time-consuming and requires special techniques and media [17]. Furthermore, the lack of official standards makes the interpretation of the results difficult. The Clinical and Laboratory Standards Institute (CLSI) has provided official breakpoints for certain antibiotics but only for human pathogen mycoplasmas [35] and the procedures and media vary according to each of the examined species [36].

Fluoroquinolones are potentially active antimicrobial agents against *M*. *hyopneumoniae* through inhibition of the bacterial DNA gyrase and topoisomerase IV enzymes [19,22]. In the present study, a broad range of MIC values was recorded with low MIC_50_ value of enrofloxacin, similarly to previous results in other European publications in the last 20 years [11,13,34]. One of the examined strains (MycSu17) was inhibited by higher enrofloxacin concentration, the MIC value against this strain exceeded the unofficial breakpoint determined by Hannan *et al*. [11]. Similar observations have already been recorded with high MIC values in Thailand (≥2 μg/ml) and in Belgium (>1 μg/ml) [13,33], which forewarns the importance of susceptibility testing before choosing antibiotics for treatment. Although MIC_50_ value of marbofloxacin against the studied strains was mostly in accordance with recent data, MIC_90_ value against the Hungarian isolates was higher than those against Belgian, Spanish and British strains (0.5–1 μg/ml) [34].

No amino acid substitutions, correlating with increased MIC values, were observed in the genes *gyrB* and *parE*, corroborating earlier publications [18]. Although single amino acid substitutions in the *parC* gene (Ser80Phe, Ser80Tyr or Asp84Asn) showed correlation with increased MIC values of fluoroquinolones in earlier publications [15,18,19], the degree of increase seems to be negligible according to the initial MIC values detected in the present study. However, definite increase of MIC values was detected when double substitutions in *parC* and *gyrA* genes were described in the examined strains. The observed effect of the double substitutions is in accordance with previous findings of Vicca et al. [18]. Various combinations of amino acid changes were detected in the examined strains containing double substitutions in the genes *gyrA* and *parC*, defining a unique combination in the outlier strain (MycSu17). Moreover, new amino acid alterations (Glu87Gly and Gly81Ala) have been described in *gyrA* gene of *M*. *hyopenumoniae* in the present study, which had been observed only in *M*. *bovis* and *M*. *gallisepticum* before [23,24]. Factors influencing the degree of the decrease of susceptibility to fluoroquinolones, such as the type of amino acid changes or mechanisms are yet to be discovered. Although initial MIC values are advised to be taken into account in the interpretation of the results of antibiotic susceptibility tests [37], correlations between the amino acid substitutions and increased final MIC values were more defined in the current examinations and better supported previous observations, which highlights the usefulness of determining final MIC values also.

The increasing susceptibility against fluoroquinolones is a notable problem, because these agents are important antibiotics for human therapy [38]. To maximize efficacy and reduce mutant selection in case of fluoroquinolones, the ratio of maximum serum concentration to the MIC (C_max_/MIC ratio) of equal or higher than 10 was proposed [39]. Marbofloxacin administered at 4 or 8 mg/kg intramuscularly resulted in 6.3 and 3.38 μg/ml C_max_ in pigs [40] respectively, resulting in maximum activity against strains with MICs of 0.625 and 0.3125 μg/ml or lower in case of the two dosages, respectively.

Tetracyclines are frequently used to control *M*. *hyopneumoniae* infections, and they act by binding to the decoding centre of the small ribosomal subunit of the bacterium [4,41]. Most of the previous publications from Europe defined similar MIC_50_ and MIC_90_ values of oxytetracycline [11,13,34]; but higher MIC_50_ and MIC_90_ values of doxycycline were described against strains originating from Spain (1 μg/ml both) and Thailand (3.12 μg/ml and 6.25 μg/ml) than against the Hungarian isolates. According to other publications supported also by our results, tetracyclines are still active against *M*. *hyopneumoniae* despite of their long-standing usage in human and veterinary medicine [32,33].

The aminoglycoside gentamicin seems to be an effective antimicrobial agent against *M*. *hyopneumoniae*, as low MIC_50_ and MIC_90_ values were observed in the present study, similarly to earlier data [13,32]. Although MIC range of the aminocyclitol spectinomycin was broad similarly to the findings of a previous Spanish study, the MIC_50_ and MIC_90_ values were higher in the present study compared to Spanish and Belgian MIC values [13,32].

Macrolides are among the most frequently used antibiotics in the swine industry to treat *M*. *hyopneumoniae* infections [4]. Both 16-membered (tylosin, tilmicosin and tylvalosin) and 15-membered (tulathromycin and gamithromycin) macrolides were effective against the studied strains. However, the MIC value of tulathromycin against the type strain was three orders of magnitude higher, than in the literature [34]. The reason of the discrepancy might be a different passage number of the type strain, or the different medium/antibiotic solution used during the test. However, the MIC value of tulathromycin against the type strain did not exceed 16 μg/ml (a possible unofficial breakpoint according to other porcine respiratory pathogens [42]) in either case. In the current study, a slight increase of MIC_50_ and MIC_90_ values of macrolides was described compared to the literature [34], and extremely high MIC values against an outlier strain (MycSu18) was noted. According to the literature, nucleotide substitutions at the bases 2057–2059 of the 23S rRNA sequence play an important role in acquired resistance to macrolides [14,19,43]. Analysis of the 23S rRNA sequence of the strain MycSu18 revealed a nucleotide substitution A2059G (*E*. *coli* numbering), which was also described in macrolide and lincosamide resistant *M*. *bovis* strains before [24]. According to the habituation study of Hannan *et al*. [12] and the high MIC values presented in this study, emergence of macrolide-resistance could be a considerable problem, which was confirmed by earlier reported results from Belgium [13], Thailand [33] and Spain [32].

Lincomycin is also active against *M*. *hyopneumoniae*, but extremely high MIC values appear every now and then [13,32,33], like the outlier strain (MycSu18) in the present study. The reason of the decreased susceptibility can be the cross-resistance with macrolides, as reported in an earlier publication, which described decreasing susceptibility against tylosin and lincomycin in strains originating from a lincomycin-treated herd [13]. The simultaneously appearing change in susceptibility may lead back to the same mode of action of macrolides and lincosamides, inhibiting bacterial protein synthesis on the 50S ribosomal subunit [44].

Pleuromutilins are important antibiotics to control *M*. *hyopneumoniae* infections through inhibiting bacterial protein synthesis [45]. According to our results and other publications, tiamulin seems to be one of the most effective antimicrobial agents against *M*. *hyopneumoniae* with low *in vitro* inhibitory concentrations [11–13,32–34]. Valnemulin is the most effective antibiotic against all of the studied strains, which supported the earlier published observations [12,32,34].

The chloramphenicol derivative florfenicol is an inhibitor of bacterial protein synthesis, used exclusively for veterinary purposes [46]. The moderate distribution of the MIC range and the relatively low MIC_50_ and MIC_90_ values of florfenicol, were similar to earlier observations from different parts of Europe and Thailand [13,33,34], and they may indicate that this antibiotic is an effective agent against *M*. *hyopneumoniae*.

*In vitro* MIC values do not necessarily correlate with the effectiveness of the antimicrobials *in vivo* and interpretation of the MIC distributions is difficult as *Mycoplasma* species with veterinary relevance do not have official clinical breakpoints [34]. Furthermore, strains with different antibiotic susceptibility can coexist within a herd [33]. PK/PD (pharmacokinetic-pharmacodynamic) analysis is an important tool to maximize *in vivo* antimicrobial activity [47,48]. Most of our results were in accordance with other results of the European region, this involves, that all the tested agents are most probably still suitable to control enzootic pneumonia. Nonetheless the results of this study may help veterinarians to choose the proper antimicrobial agent against *M*. *hyopneumoniae*. Although the isolation of *M*. *hyopneumoniae* strains is a time-consuming and fastidious process, the regularly accomplished antibiotic susceptibility testing of the swine herds should enable appropriate antibiotic usage during treatment. Furthermore, the development of PCR-based susceptibility tests based on SNPs correlating with changes in the MIC values, could improve diagnostics and treatment, similarly to antibiotic susceptibility testing in *M*. *bovis* [24].

## Conclusion

This study provided current and relevant information about the antibiotic susceptibility profiles of *M*. *hyopneumoniae* strains circulating in Hungary and surrounding countries. Low MIC values of all the tested antibiotics were described against most of the studied *M*. *hyopneumoniae* strains, and the lowest MIC values were found in case of gentamicin, tylosin, tylvalosin, lincomycin, tiamulin and valnemulin. In certain cases, high MIC values of fluoroquinolones (MycSu17) or macrolides and lincomycin (MycSu18) were observed. Single or double amino acid substitutions in the genes *gyrA* (Gly81Ala, Ala83Val, Glu87Gly), *parC* (Ser80Phe, Ser80Tyr, Asp84Asn) and a SNP in the 23S rRNA sequence (A2059G) were also detected correlating with decreased antibiotic susceptibilities. Macrolides and fluoroquinolones are frequently used empirically as a first choice for the management of mycoplasmoses in livestock in Europe. The regular testing of the sensitivity profile of *M*. *hyopneumoniae*, the determination of herd specific MICs would promote the use of less critical antibacterials (e.g.: florfenicol, tetracyclines, pleuromutilins), and might contribute to the preservation of the critically important antibiotics (macrolides and fluoroquinolones) both for veterinary and human medicine.

## Supporting information

S1 Table Background data of *M*. *hyopneumoniae* strains and initial minimum inhibitory concentration (MIC) values (μg/ml) of 15 antimicrobials against the strains used in the study. Isolation data (Sample ID, Herd of origin and Date of isolation) and MIC values of enrofloxacin (EFX), marbofloxacin (MFX), oxytetracycline (OTC), doxycycline (DX), gentamicin (GTC), spectinomycin (SPC), tylosin (TYL), tilmicosin (TIL), tylvalosin (TVN), gamithromycin (GTM), tulathromycin (TTM), tiamulin (TIA), valnemulin (VAL), lincomycin (LCM) and florfenicol (FFC) are presented. Abbreviations for herd of origin are: H-Hungary, CZ-Czech Republic, SK-Slovakia.
(DOCX)

S2 Table Background data of *M*. *hyopneumoniae* strains and final minimum inhibitory concentration (MIC) values (μg/ml) of 15 antimicrobials against the strains used in the study. Isolation data (Sample ID, Herd of origin and Date of isolation) and MIC values of enrofloxacin (EFX), marbofloxacin (MFX), oxytetracycline (OTC), doxycycline (DX), gentamicin (GTC), spectinomycin (SPC), tylosin (TYL), tilmicosin (TIL), tylvalosin (TVN), gamithromycin (GTM), tulathromycin (TTM), tiamulin (TIA), valnemulin (VAL), lincomycin (LCM) and florfenicol (FFC) are presented. Abbreviations for herd of origin are: H-Hungary, CZ-Czech Republic, SK-Slovakia.
(DOCX)

S3 Table Initial and final minimum inhibitory concentration (MIC) ranges (μg/ml) of fluoroquinolones, macrolides and lincomycin against the examined *M*. *hyopneumoniae* isolates with the amino acid substitutions in the *gyrA* and *parC* genes and nucleotide substitutions in the 23S rRNA sequence. (DOCX)
