# Supplementary material for: Antibiotic susceptibility testing of Mycoplasma hyopneumoniae field isolates from Central Europe for fifteen antibiotics by microbroth dilution method
Source: PLoS One. 2018 Dec 11;13(12):e0209030. doi: 10.1371/journal.pone.0209030 (PMC6289410; doi:10.1371/journal.pone.0209030)
Supplement: S2 Table — Isolation data (Sample ID, Herd of origin and Date of isolation) and MIC values of enrofloxacin (EFX), marbofloxacin (MFX), oxytetracycline (OTC), doxycycline (DX), gentamicin (GTC), spectinomycin (SPC), tylosin (TYL), tilmicosin (TIL), tylvalosin (TVN), gamithromycin (GTM), tulathromycin (TTM), tiamulin (TIA), valnemulin (VAL), lincomycin (LCM) and florfenicol (FFC) are presented. Abbreviations for herd of origin are: H-Hungary, CZ-Czech Republic, SK-Slovakia. (DOCX) [file pone.0209030.s002.docx]

| Data of isolation | | | Final MIC values (µg/ml) | | | | | | | | | | | | | | |
| --- | --- | --- | --- | --- | --- | --- | --- | --- | --- | --- | --- | --- | --- | --- | --- | --- | --- |
| Sample ID | Herd of origin | Date of isolation | EFX | MFX | OTC | DX | GTC | SPC | TYL | TIL | TVN | GTM | TTM | LCM | TIA | VAL | FFC |
| MycSu1 | H, Hajduszoboszlo | 2015 | 0.312 | 1.25 | 4 | 0.312 | 2 | 4 | 0.5 | 8 | ≤0.25 | 4 | 2 | 1 | 0.156 | ≤0.039 | 2 |
| MycSu2 | H, Hajduszoboszlo | 2015 | 0.312 | 1.25 | 4 | 0.312 | 1 | 8 | 0.5 | 4 | ≤0.25 | 4 | 4 | 1 | 0.156 | ≤0.039 | 2 |
| MycSu3 | H, Nagyhegyes | 2015 | 0.078 | 0.156 | 4 | 0.625 | 1 | 2 | 0.5 | 4 | ≤0.25 | 4 | 2 | 0.5 | 0.156 | ≤0.039 | 2 |
| MycSu4 | H, Nagyhegyes | 2015 | ≤0.039 | ≤0.039 | 8 | 1.25 | 0.5 | 4 | 0.5 | 8 | ≤0.25 | 4 | 2 | 0.5 | 0.156 | ≤0.039 | 2 |
| MycSu5 | H, Tata | 2015 | ≤0.039 | 0.078 | 32 | 1.25 | 1 | 4 | ≤0.25 | 4 | ≤0.25 | 4 | 1 | 0.5 | 0.156 | ≤0.039 | 2 |
| MycSu6 | H, Tata | 2015 | 0.078 | 0.078 | 8 | 0.625 | 1 | 2 | 0.5 | 8 | ≤0.25 | 4 | 2 | 1 | 0.156 | ≤0.039 | 2 |
| MycSu7 | H, Mezotur | 2015 | 0.078 | 0.078 | 1 | 0.156 | 2 | 4 | 0.5 | 4 | ≤0.25 | 2 | 2 | 0.5 | 0.156 | ≤0.039 | 4 |
| MycSu8 | H, Mezotur | 2015 | 0.312 | 1.25 | 4 | 0.312 | 0.5 | 4 | 0.5 | 4 | ≤0.25 | 2 | 1 | 0.5 | 0.156 | ≤0.039 | 2 |
| MycSu9 | H, Hajdunanas | 2015 | ≤0.039 | ≤0.039 | 0.5 | 0.156 | 1 | 4 | ≤0.25 | 8 | ≤0.25 | 2 | 1 | 0.5 | 0.078 | ≤0.039 | 2 |
| MycSu10 | H, Bekescsaba | 2015 | 0.312 | 1.25 | 4 | 0.312 | 1 | 2 | ≤0.25 | 4 | ≤0.25 | 4 | 1 | 0.5 | 0.156 | ≤0.039 | 2 |
| MycSu11 | H, Bekescsaba | 2015 | 0.312 | 1.25 | 8 | 0.625 | 1 | 4 | 0.5 | 8 | ≤0.25 | 4 | 2 | 1 | 0.156 | ≤0.039 | 4 |
| MycSu12 | H, Dombegyhaz | 2015 | 2.5 | 5 | 2 | 0.312 | 2 | 4 | 0.5 | 8 | ≤0.25 | 4 | 2 | 0.5 | 0.156 | ≤0.039 | 2 |
| MycSu13 | H, Rabaszentandras | 2015 | 0.312 | 1.25 | 4 | 0.312 | 1 | 4 | 0.5 | 4 | ≤0.25 | 2 | 1 | 1 | 0.156 | ≤0.039 | 2 |
| MycSu14 | H, Csikostottos | 2015 | 0.078 | 0.078 | 0.5 | 0.078 | 2 | 8 | 0.5 | 8 | ≤0.25 | 8 | 2 | 1 | 0.156 | ≤0.039 | 2 |
| MycSu15 | H, Bacsalmas | 2015 | 2.5 | 5 | 16 | 2.5 | 0.5 | 2 | ≤0.25 | 8 | ≤0.25 | 2 | 1 | ≤0.25 | 0.078 | ≤0.039 | 2 |
| MycSu16 | H, Bacsalmas | 2015 | 2.5 | 5 | 16 | 2.5 | 0.5 | 4 | ≤0.25 | 8 | ≤0.25 | 1 | 1 | 0.5 | 0.156 | ≤0.039 | 2 |
| MycSu17 | H, Palhalma | 2016 | 5 | 10 | 4 | 0.312 | 0.5 | 2 | 0.5 | 8 | ≤0.25 | 8 | 2 | 1 | 0.156 | ≤0.039 | 2 |
| MycSu18 | H, Bacsalmas | 2016 | 2.5 | 5 | 8 | 1.25 | 0.5 | 2 | 64 | >64 | 8 | >64 | >64 | >64 | 0.312 | ≤0.039 | 2 |
| MycSu19 | H, Labod | 2016 | 0.312 | 1.25 | 16 | 1.25 | 1 | 4 | ≤0.25 | 4 | ≤0.25 | 2 | 1 | 1 | 0.156 | ≤0.039 | 2 |
| MycSu20 | CZ, no data | 2016 | 2.5 | 5 | 32 | 1.25 | 0.5 | 4 | 0.5 | 8 | ≤0.25 | 4 | 1 | 0.5 | 0.156 | ≤0.039 | 2 |
| MycSu33 | H, Labod | 2016 | 0.312 | 1.25 | 4 | 0.625 | 0.5 | 4 | ≤0.25 | 8 | ≤0.25 | 4 | 2 | 1 | 0.156 | ≤0.039 | 2 |
| MycSu34 | H, Oroshaza | 2016 | ≤0.039 | ≤0.039 | 8 | 0.625 | 0.5 | 2 | 0.5 | 8 | ≤0.25 | 4 | 2 | 0.5 | 0.156 | ≤0.039 | 2 |
| MycSu37 | H, Csemo | 2016 | 0.625 | 2.5 | 16 | 1.25 | 0.5 | 2 | 0.5 | 4 | ≤0.25 | 4 | 2 | 1 | 0.156 | ≤0.039 | 1 |
| MycSu39 | H, Sellye | 2016 | 0.312 | 1.25 | 2 | 0.312 | 0.5 | 4 | ≤0.25 | 4 | ≤0.25 | 2 | 0.5 | 1 | 0.078 | ≤0.039 | 2 |
| MycSu40 | H, Felsobabad | 2016 | ≤0.039 | 0.078 | 8 | 1.25 | 0.5 | 4 | 1 | 16 | ≤0.25 | 16 | 2 | 0.5 | 0.156 | ≤0.039 | 4 |
| MycSu41 | H, Mezotur | 2016 | 5 | 5 | 8 | 1.25 | 0.5 | 1 | ≤0.25 | 4 | ≤0.25 | 2 | 1 | 1 | 0.078 | ≤0.039 | 1 |
| MycSu42 | H, Mesterszallas | 2016 | ≤0.039 | 0.078 | 8 | 0.625 | 0.5 | 2 | 0.5 | 16 | ≤0.25 | 8 | 4 | 1 | 0.156 | ≤0.039 | 4 |
| MycSu43 | SK, no data | 2016 | ≤0.039 | ≤0.039 | 32 | 2.5 | 1 | 4 | ≤0.25 | 16 | ≤0.25 | 8 | 2 | 1 | 0.156 | ≤0.039 | 2 |
| MycSu44 | SK, no data | 2016 | 2.5 | 1.25 | 1 | 0.156 | 1 | 4 | 0.5 | 16 | ≤0.25 | 4 | 2 | 0.5 | 0.156 | ≤0.039 | 2 |
| MycSu45 | H, Dobrokoz | 2016 | 2.5 | 5 | 4 | 0.625 | 2 | 8 | >0.25 | 8 | ≤0.25 | 4 | 2 | 1 | 0.156 | ≤0.039 | 2 |
| MycSu46 | H, Szentes | 2016 | 0.312 | 1.25 | 4 | 0.625 | 0.5 | 2 | 0.5 | 16 | ≤0.25 | 8 | 2 | 1 | 0.156 | ≤0.039 | 2 |
| MycSu47 | H, Lovasbereny | 2016 | ≤0.039 | 0.156 | 0.5 | 0.078 | 1 | 2 | 0.5 | 32 | ≤0.25 | 8 | 4 | 1 | 0.156 | ≤0.039 | 4 |
| MycSu49 | H, Felsobabad | 2016 | ≤0.039 | 0.078 | 8 | 0.625 | 0.5 | 4 | 0.5 | 8 | ≤0.25 | 4 | 2 | 0.5 | 0.156 | ≤0.039 | 2 |
| MycSu50 | H, Fegyvernek | 2016 | 5 | 5 | 4 | 0.625 | 0.5 | 4 | 0.5 | 8 | ≤0.25 | 8 | 2 | 0.5 | 0.156 | ≤0.039 | 4 |
| MycSu52 | H, Baracska | 2016 | 0.078 | 0.078 | 4 | 0.625 | 0.5 | 2 | 0.5 | 8 | ≤0.25 | 4 | 2 | 0.5 | 0.156 | ≤0.039 | 2 |
| MycSu53 | H, Ocsa | 2016 | 0.625 | 1.25 | 4 | 0.312 | 1 | 4 | ≤0.25 | 4 | ≤0.25 | 4 | 1 | ≤0.25 | 0.156 | ≤0.039 | 2 |
| MycSu70 | H, Fabiansebestyen | 2016 | ≤0.039 | 0.078 | 32 | 2.5 | 2 | 4 | ≤0.25 | 2 | ≤0.25 | 2 | 0.5 | 1 | 0.078 | ≤0.039 | 4 |
| MycSu79 | H, Papa | 2016 | 0.312 | 1.25 | 4 | 0.625 | 0.5 | 2 | ≤0.25 | 16 | ≤0.25 | 4 | 2 | 0.5 | 0.156 | ≤0.039 | 4 |
| MycSu80 | H, Nadudvar | 2016 | 0.625 | 2.5 | 4 | 0.625 | 1 | 2 | 0.5 | 4 | ≤0.25 | 4 | 2 | 1 | 0.156 | ≤0.039 | 2 |
| MycSu81 | H, Nadudvar | 2016 | 0.625 | 1.25 | 4 | 0.312 | 1 | 2 | 0.5 | 8 | ≤0.25 | 4 | 1 | 0.5 | 0.156 | ≤0.039 | 4 |
| MycSu82 | H, Nadudvar | 2016 | 0.625 | 1.25 | 4 | 0.625 | 1 | 2 | 0.5 | 8 | ≤0.25 | 4 | 2 | 0.5 | 0.156 | ≤0.039 | 4 |
| MycSu83 | SK, no data | 2016 | ≤0.039 | 0.078 | 4 | 0.312 | 1 | 4 | 0.5 | 8 | ≤0.25 | 8 | 1 | 0.5 | 0.156 | ≤0.039 | 2 |
| MycSu84 | H, Osli | 2016 | ≤0.039 | 0.078 | 16 | 1.25 | 1 | 2 | ≤0.25 | 4 | ≤0.25 | 2 | 2 | 0.5 | 0.156 | ≤0.039 | 2 |
| MycSu85 | H, Devavanya | 2016 | 0.078 | 0.078 | 16 | 2.5 | 1 | 2 | 0.5 | 8 | ≤0.25 | 4 | 1 | 1 | 0.156 | ≤0.039 | 2 |
