# Supplementary material for: Antibiotic susceptibility testing of Mycoplasma hyopneumoniae field isolates from Central Europe for fifteen antibiotics by microbroth dilution method
Source: PLoS One. 2018 Dec 11;13(12):e0209030. doi: 10.1371/journal.pone.0209030 (PMC6289410; doi:10.1371/journal.pone.0209030)
Supplement: S3 Table — (DOCX) [file pone.0209030.s003.docx]

| Sample ID | Range of MIC values (µg/ml) | | | | | | | | Substitutions | | |
| --- | --- | --- | --- | --- | --- | --- | --- | --- | --- | --- | --- |
|  | Fluoroquinolones | | 16-membered macrolides | | 15-membered macrolides | | Lincomycin | | *gyrA* | *parC* | 23S rRNA |
|  | initial | final | initial | final | initial | final | initial | final |  |  |  |
| MycSu4 | ≤0.039 | ≤0.039 | ≤0.25-4 | ≤0.25-8 | ≤0.25-0.5 | 2-4 | ≤0.25 | 0.5 |  |  |  |
| MycSu9 | ≤0.039 | ≤0.039 | ≤0.25-2 | ≤0.25-8 | ≤0.25-0.5 | 1-2 | ≤0.25 | 0.5 |  |  |  |
| MycSu34 | ≤0.039 | ≤0.039 | ≤0.25-4 | ≤0.25-8 | ≤0.25-0.5 | 2-4 | ≤0.25 | 0.5 |  |  |  |
| MycSu43 | ≤0.039 | ≤0.039 | ≤0.25-8 | ≤0.25-16 | 1 | 2-8 | ≤0.25 | 1 |  |  |  |
| MycSu5 | ≤0.039 | ≤0.039-0.078 | ≤0.25-2 | ≤0.25-4 | ≤0.25-0.5 | 1-4 | ≤0.25 | 0.5 |  |  |  |
| MycSu40 | ≤0.039 | ≤0.039-0.078 | ≤0.25-4 | ≤0.25-16 | 1-2 | 2-16 | ≤0.25 | 0.5 |  |  |  |
| MycSu42 | ≤0.039 | ≤0.039-0.078 | ≤0.25-4 | ≤0.25-16 | 2-4 | 4-8 | 0.5 | 1 |  |  |  |
| MycSu49 | ≤0.039 | ≤0.039-0.078 | ≤0.25-4 | ≤0.25-8 | 0.5-2 | 2-4 | ≤0.25 | 0.5 |  |  |  |
| MycSu70 | ≤0.039 | ≤0.039-0.078 | ≤0.25-1 | ≤0.25-2 | ≤0.25 | 0.5-2 | ≤0.25 | 1 |  |  |  |
| MycSu83 | ≤0.039 | ≤0.039-0.078 | ≤0.25-4 | ≤0.25-8 | 1-2 | 1-8 | ≤0.25 | 0.5 |  |  |  |
| MycSu84 | ≤0.039 | ≤0.039-0.078 | ≤0.25-2 | ≤0.25-4 | 0.5 | 2 | ≤0.25 | 0.5 |  |  |  |
| MycSu47 | ≤0.039 | ≤0.039-0.156 | ≤0.25-2 | ≤0.25-32 | 2 | 4-8 | ≤0.25 | 1 |  |  |  |
| MycSu6 | ≤0.039 | 0.078 | ≤0.25-2 | ≤0.25-8 | 0.5-1 | 2-4 | ≤0.25 | 1 |  |  |  |
| MycSu7 | ≤0.039 | 0.078 | ≤0.25-0.5 | ≤0.25-4 | ≤0.25 | 2 | ≤0.25 | 0.5 |  |  |  |
| MycSu14 | ≤0.039 | 0.078 | ≤0.25-2 | ≤0.25-8 | 0.5-1 | 2-8 | ≤0.25 | 1 |  |  |  |
| MycSu52 | ≤0.039 | 0.078 | ≤0.25-2 | ≤0.25-8 | 1 | 2-4 | ≤0.25 | 0.5 |  |  |  |
| MycSu85 | ≤0.039 | 0.078 | ≤0.25-2 | ≤0.25-8 | 0.5 | 1-4 | ≤0.25 | 1 |  |  |  |
| MycSu3 | ≤0.039 | 0.078-0.156 | ≤0.25-2 | ≤0.25-4 | 1 | 2-4 | ≤0.25 | 0.5 |  |  |  |
| MycSu2 | ≤0.039 | 0.312-1.25 | ≤0.25-4 | ≤0.25-4 | 2 | 4 | 0.5 | 1 |  | Ser80Phe |  |
| MycSu10 | ≤0.039 | 0.312-1.25 | ≤0.25-2 | ≤0.25-4 | 0.5-1 | 1-4 | ≤0.25 | 0.5 |  | Ser80Phe |  |
| MycSu11 | ≤0.039 | 0.312-1.25 | ≤0.25-2 | ≤0.25-8 | 0.5-1 | 2-4 | ≤0.25 | 1 |  | Ser80Phe |  |
| MycSu39 | ≤0.039 | 0.312-1.25 | ≤0.25-2 | ≤0.25-4 | ≤0.25 | 0.5-2 | ≤0.25 | 1 |  | Asp84Asn |  |
| MycSu33 | ≤0.039-0.078 | 0.312-1.25 | ≤0.25-2 | ≤0.25-8 | ≤0.25-0.5 | 2-4 | ≤0.25 | 1 |  | Ser80Phe |  |
| MycSu79 | ≤0.039-0.078 | 0.312-1.25 | ≤0.25-4 | ≤0.25-16 | 1 | 2-4 | ≤0.25 | 0.5 |  | Ser80Tyr |  |
| MycSu1 | ≤0.039-0.312 | 0.312-1.25 | ≤0.25-4 | ≤0.25-8 | 1 | 2-4 | ≤0.25 | 1 |  | Ser80Phe |  |
| MycSu13 | ≤0.039-0.312 | 0.312-1.25 | ≤0.25-2 | ≤0.25-4 | 1 | 1-2 | ≤0.25 | 1 |  | Ser80Phe |  |
| MycSu8 | 0.078-0.625 | 0.312-1.25 | ≤0.25-2 | ≤0.25-4 | 0.5 | 1-2 | ≤0.25 | 0.5 |  | Ser80Phe |  |
| MycSu46 | 0.078-0.625 | 0.312-1.25 | ≤0.25-4 | ≤0.25-16 | 1-2 | 2-8 | ≤0.25 | 1 |  | Ser80Phe |  |
| MycSu19 | 0.156-0.625 | 0.312-1.25 | ≤0.25-1 | ≤0.25-4 | 0.5 | 1-2 | ≤0.25 | 1 |  | Ser80Phe |  |
| MycSu53 | ≤0.039 | 0.625-1.25 | ≤0.25 | ≤0.25-4 | ≤0.25-0.5 | 1-4 | ≤0.25 | ≤0.25 |  | Ser80Phe |  |
| MycSu81 | 0.156-0.625 | 0.625-1.25 | ≤0.25-2 | ≤0.25-8 | 1 | 1-4 | ≤0.25 | 0.5 |  | Ser80Phe |  |
| MycSu82 | 0.156-0.625 | 0.625-1.25 | ≤0.25-4 | ≤0.25-8 | 0.5 | 2-4 | ≤0.25 | 0.5 |  | Ser80Phe |  |
| MycSu37 | ≤0.039-0.312 | 0.625-2.5 | ≤0.25 | ≤0.25-4 | ≤0.25-1 | 2-4 | ≤0.25 | 1 |  | Ser80Phe |  |
| MycSu80 | 0.312-0.625 | 0.625-2.5 | ≤0.25-2 | ≤0.25-4 | 0.5 | 2-4 | ≤0.25 | 1 |  | Ser80Phe |  |
| MycSu44 | 0.625 | 1.25-2.5 | ≤0.25-4 | ≤0.25-16 | ≤0.25-0.5 | 2-4 | ≤0.25 | 0.5 | Ala83Val | Asp84Asn |  |
| MycSu20 | 0.156-0.625 | 2.5-5 | ≤0.25-4 | ≤0.25-8 | 1 | 1-4 | ≤0.25 | 0.5 | Ala83Val | Ser80Tyr |  |
| MycSu12 | 0.625-1.25 | 2.5-5 | ≤0.25-1 | ≤0.25-8 | 1 | 2-4 | ≤0.25 | 0.5 | Ala83Val | Ser80Tyr |  |
| MycSu15 | 1.25-2.5 | 2.5-5 | ≤0.25-4 | ≤0.25-8 | ≤0.25-1 | 1-2 | ≤0.25 | ≤0.25 | Gly81Ala | Ser80Tyr |  |
| MycSu16 | 1.25-2.5 | 2.5-5 | ≤0.25-2 | ≤0.25-8 | ≤0.25 | 1 | ≤0.25 | 0.5 | Gly81Ala | Ser80Tyr |  |
| MycSu18 | 1.25-2.5 | 2.5-5 | 32>64 | 8->64 | 64->64 | >64 | >64 | >64 | Gly81Ala | Ser80Tyr | A2059G |
| MycSu45 | 1.25-2.5 | 2.5-5 | ≤0.25 | ≤0.25-8 | ≤0.25 | 2-4 | ≤0.25 | 1 | Glu87Gly | Ser80Phe |  |
| MycSu41 | 1.25 | 5 | ≤0.25-1 | ≤0.25-4 | ≤0.25 | 1-2 | ≤0.25 | 1 | Gly81Ala | Ser80Tyr |  |
| MycSu50 | 1.25-2.5 | 5 | ≤0.25 | ≤0.25-8 | ≤0.25 | 2-8 | ≤0.25 | 0.5 | Glu87Gly | Ser80Tyr |  |
| MycSu17 | 2.5-5 | 5-10 | ≤0.25-4 | ≤0.25-8 | 0.5-1 | 2-8 | 0.5 | 1 | Ala83Val | Ser80Phe |  |
